# Influence of transcutaneous vagus nerve stimulation on cardiac vagal activity: Not different from sham stimulation and no effect of stimulation intensity

**DOI:** 10.1371/journal.pone.0223848

**Published:** 2019-10-11

**Authors:** Uirassu Borges, Sylvain Laborde, Markus Raab

**Affiliations:** 1 German Sport University Cologne, Germany; 2 Normandie University, France; 3 London South Bank University, England; Heidelberg University, GERMANY

## Abstract

The present study investigated the effects of transcutaneous vagus nerve stimulation on cardiac vagal activity, the activity of the vagus nerve regulating cardiac functioning. We applied stimulation on the left cymba conchae and tested the effects of different stimulation intensities on a vagally-mediated heart rate variability pagerameter (i.e., the root mean square of successive differences) as well as on subjective ratings of strength of perceived stimulation intensity and unpleasantness due to the stimulation. Three experiments (within-subject designs, *M* = 61 healthy participants each) were carried out: In Experiment 1, to choose one fixed stimulation intensity for the subsequent studies, we compared three preset stimulation intensities (i.e., 0.5, 1.0 and 1.5 mA) with each other. In Experiment 2, we compared the set stimulation method with the free stimulation method, in which the participants were instructed to freely choose an intensity. In Experiment 3, to control for placebo effects, we compared both methods (i.e., set stimulation vs. free stimulation) with their respective sham stimulations. In the three experiments, an increase of cardiac vagal activity was found from resting to the stimulation phases. However, this increase in cardiac vagal activity was not dependent on stimulation intensity (Experiment 1), the method used to stimulate (i.e., set vs. free; Experiment 2), or whether stimulation was active or sham (Experiment 3). This pattern of results was solidly supported by Bayesian estimations. On the subjective level, higher stimulation intensities were perceived as significantly stronger and a stronger stimulation was generally also perceived as more unpleasant. The results suggest that cardiac vagal activity may be similarly influenced by afferent vagal stimuli triggered by active and sham stimulation with different stimulation intensities. Potential explanations for these findings and its implications for future research with tVNS are discussed.

## Introduction

Transcutaneous vagus nerve stimulation (tVNS) is a noninvasive technology used to electrically modulate vagal activity and consequently brain activity via afferent vagal pathways [1]. Because of its safety and the absence of major side effects [1,2], tVNS has been applied in both research and therapy as a medical treatment tool. In recent years, this research field has seen a noteworthy growth through studies investigating how tVNS positively affects cognitive [3], affective [4], and neurophysiological [5] processes. However, because of the novelty of this technology and the absence of standards regarding stimulation protocol, the tVNS-related stimulation parameters have not been used consistently in research [6], which impedes the comparability of such studies. Thus, understanding both the action mechanism of this neuromodulation tool and the processes yielded by the stimulation seems to be crucial. The present work addressed this issue with a focus on stimulation intensity, also known as amplitude, as a changeable stimulation parameter across three experiments and investigated the influence of tVNS on a psychophysiological marker, heart rate variability (HRV).

Essentially, tVNS acts on the afferent auricular branch of the vagus nerve through electrodes placed on the skin of the left ear. Its placement allows for a sham stimulation, which has the same characteristics as normal tVNS, but instead of the electrodes being attached to the cymba conchae, they are attached to the earlobe. The earlobe is thought to be free of vagal innervation [7]. The tVNS’ mechanism of action can be explained by considering the neuroanatomical pathways of the vagus nerve. The electrical signal, starting in the auricular branch of the vagus nerve, reaches the nucleus tractus solitarius, which is a crucial structure that projects to a variety of brain areas, for instance the locus coeruleus [1]. Locus coeruleus is the primary source of norepinephrine in the brain [8]. The noradrenergic supply includes cortical regions such as the anterior cingulate cortex and the prefrontal cortex [9]. An increase in prefrontal activity leads to an increase in parasympathetic nervous system activity and to a decrease in sympathetic activity, since the prefrontal cortex is thought to exert a tonic inhibitory control on the sympathetic nervous system [10]. According to the neurovisceral integration model [11], the prefrontal cortex controls cardiac vagal activity, the activity of the vagus nerve regulating cardiac functioning [12], via top-down mechanisms, and is thought to be also positively linked to cognitive processes such as executive functioning [11]. Consequently, higher cardiac vagal activity is linked to the optimal activation of neural networks underlying the effectiveness of prefrontal activity [10]. Given this regulatory role of the prefrontal cortex on cardiac vagal activity and that the tVNS signal is sent afferently to the prefrontal cortex via the auricular branch of the vagus nerve, cardiac vagal activity may be affected by tVNS [13]. It has been discussed that tVNS targets three neurotransmitters, namely, norepinephrine, gamma-aminobutyric acid, and acetylcholine, which are thought to be directly involved in cognitive functioning [14]. As mentioned above, an increase in cardiac vagal activity can indicate that tVNS, when applied on the left auricular branch of the vagus nerve, is afferently sending a signal to the prefrontal cortex, therefore positively affecting cognitive functioning. Thus, measuring cardiac vagal activity during tVNS may lead to a better understanding of the physiological pathways behind specific cognitive processes.

An array of studies using tVNS performs electrode placement on the left side of the ear in order to control for cardiac side effects [3,15–20]. This is based on previous research stating that efferent vagal fibers to the heart are located on the right side, whereas the left vagus nerve mainly consists of afferent vagal fibers [21]. Since the present study aims at addressing the effect of tVNS on cardiac vagal activity through the pathway explained previously and not by pathways to which the prefrontal cortex seems to be less related, we performed the stimulation on the left ear.

In contrast to sham stimulation, tVNS produces a significant activation of central vagal projections, as shown by functional magnetic resonance imaging (fMRI) studies [16,22–25] of, for example, the nucleus tractus solitarius [16,24] and the locus coeruleus, [23,25] as well as the left prefrontal cortex and cingulate areas [22]. However, these studies used very different stimulation parameters, for example, differing in electrode placement areas on the ear, pulse width, frequency, and on–off cycle. Possibly because of the use of different stimulation parameters, these studies had heterogeneous results, with different brain areas being found to be related to tVNS [22]. Using HRV-related parameters to measure autonomic balance, Clancy and colleagues [26] showed that tVNS can increase parasympathetic activity and simultaneously suppress sympathetic activity. However, this positive effect of tVNS on vagally related HRV could not be clearly shown in other studies [4,27–29]. These contradictory results might be, again, due to the use of different stimulation parameters in these studies, for instance, the use of different devices and consequently different positioning of the electrodes on the ear, resulting in a lack of comparability across results. Varying stimulation intensities might have also played a role in these divergent findings.

Given the substantial heterogeneity in tVNS literature regarding choice of stimulation parameters, the lack of knowledge about optimal stimulation parameters can be seen as a general limitation in this field [4,22,26]. Likewise, the consideration of certain HRV parameters to measure the effects of tVNS on cardiac activity may render their interpretation difficult. For instance, Clancy [26] showed that tVNS can evoke a decrease in the ratio between low and high frequencies of HRV (LF/HF), with this result being interpreted as a shift in cardiac autonomic function toward parasympathetic dominance. However, it is noteworthy that the role of this HRV parameter in depicting sympathovagal balance has recently been discarded, so no real physiological conclusion can be drawn from this finding [30]. Recently, Badran and colleagues [6] systematically tested the effect of variations in pulse width and frequency on heart rate and found that a pulse width of 500 μs, if combined with a frequency of 10 Hz, provoked the strongest decrease in heart rate compared to other parameter combinations. Nonetheless, heart rate represents the result of mixed inputs from the sympathetic and parasympathetic (vagus) nerves [31,32]. Thus, this finding cannot be linked specifically to cardiac vagal activity. Consequently, investigating the effect of tVNS parameters on valid indicators of cardiac vagal activity remains a research gap to be filled [33].

Stimulation intensity, also known as amplitude, varies highly by experimental protocol in tVNS studies. This parameter is also the only one that can be changed in the presently most commonly used tVNS device in cognitive research, NEMOS by Cerbomed (Erlangen, Germany). To date, there is no standard protocol for tVNS to systematically set this parameter. Two main methods have been identified in the literature to set stimulation intensity: what we call the set stimulation method and the free stimulation method. In the set stimulation method, the stimulation intensity is determined by the experimenters beforehand and therefore does not vary individually across the experiment. The predetermined intensity has often been set at 0.5 mA [3,20,34], and the choice of this intensity is justified as being recommended by another study [23]. However, no mention of 0.5 mA could be found in this original work. In the free stimulation method, participants are instructed to freely choose the highest subjectively comfortable stimulation intensity below the discomfort threshold [17,18,27,35].

The effects of tVNS are thought to exhibit an inverted U-shaped relationship concerning stimulation intensity, so that, similar to the Yerkes–Dodson principle [36], an intermediate stimulation intensity provokes the strongest effect [1,14]. One explanation for this pattern is that a low stimulation intensity may not be enough to activate the afferent vagal pathway, whereas a high stimulation intensity can cause discomfort. Discomfort is thought to negatively impact HRV parameters [37]. Another possible explanation is that participants experience control over the stimulation intensity in the free compared to the set stimulation method. Feelings of increased control can affect anxiety and pain, as well as the willingness to tolerate discomfort. Although there is evidence of this inverted U-shaped relationship for invasive (cervical) VNS regarding memory retention [38] and cortical plasticity [39], there is still a lack of evidence for tVNS regarding cardiac regulation, making this idea merely speculative for cardiac vagal activity. Insights on this potentially existing inverted U-shaped relationship for tVNS with its expected outcomes could be important for optimizing its effects and eventually for improving safety. Therefore, the existence of the inverted U-shaped relationship should be further investigated [1,14].

In summary, discrepancies appear in the literature regarding tVNS stimulation intensity. The present study goes beyond existing research on tVNS by focusing on this issue and systematically testing different stimulation intensity settings for tVNS and their effects on cardiac vagal activity, as measured using HRV parameters, addressing both set and free stimulation methods for determining stimulation intensity. The objectives of the present work were threefold: first, to investigate the effects of tVNS on cardiac vagal activity dependent on stimulation intensity; second, to find the optimal way to choose a stimulation intensity that provokes the strongest increase in cardiac vagal activity; and third, to examine the differences in how stimulation methods are subjectively rated in terms of perceived strength and (un)pleasantness. To answer these questions, we designed three experiments that addressed different stimulation intensities as well as different methods to determine stimulation intensity. Across all three experiments, (H1) we expected cardiac vagal activity to increase during tVNS compared to baseline measurements. Specifically, for Experiment 1 we hypothesized (H2) that when using the set stimulation method, the intermediate stimulation intensity would increase cardiac vagal activity more than both the weaker and the stronger stimulation intensity, in accordance with the Yerkes–Dodson principle [36]. For Experiments 2 and 3, we expected (H3) the free stimulation method to evoke a higher increase in cardiac vagal activity compared to the set stimulation method, given that an unpleasant stimulation intensity can be better avoided by means of a subjectively more suitable self-chosen intensity. Finally, for Experiment 3, we expected (H4) tVNS to provoke higher cardiac vagal activity compared to sham stimulation.

## General method

We conducted three single-blind experiments with a within-subject design, as recommended by Quintana and Heathers [40], to address the high interindividual variation and the complex interactions influencing HRV. Aiming at choosing one fixed stimulation intensity to be used in Experiments 2 and 3, in Experiment 1 we focused on three different fixed stimulation intensities, allowing us to explore the set stimulation method and its effects on cardiac vagal activity. In Experiment 2, focusing on the two most common methods to determine stimulation intensity in tVNS research, we compared the set stimulation method with the free stimulation method regarding their effect on cardiac vagal activity. To shorten the experiments’ length and to avoid an eventual positive effect of habituation on cardiac vagal activity, both Experiments 1 and 2 were done without a sham condition. In Experiment 3, to control for confounding effects such as a placebo effect, we compared both methods with their respective sham stimulations regarding cardiac vagal activity.

### Participants

An a priori G*Power calculation [41] was carried out to estimate the sample size required for the three experiments. On the basis of previously published [26] recalculated effect sizes of the LF/HF ratio, we expected small to medium effect sizes for the effects of tVNS on HRV. The calculation for a repeated measure analysis of variance (ANOVA) with within-subject factors, *f* = .17, α = .05, power = .80, estimated that 60 participants were required for each experiment. Anticipating possible exclusions after data cleaning, we recruited on average 65 participants for each study.

The sample consisted of healthy sport science students at the local university. Participants were eligible if they were free of cardiovascular or neurological diseases or major mental conditions and were not pregnant at the time of the experiment. They were asked not to smoke, exercise, or consume food, alcohol, or caffeine for at least 2 h before participation. These potentially confounding variables as well as tVNS safety-related questions were assessed by means of an adapted version of the questionnaire for HRV psychophysiological experiments developed by Laborde et al. [33]. None of the participants had experienced tVNS prior to this study. A new sample was tested in each experiment and participants gave written informed consent prior to the experiment. All experiments were approved by the ethical committee of the German Sport University Cologne (ethics approval numbers 158/2016, 078/2017 and 019/2018, respectively).

### Materials and methods

#### Transcutaneous vagus nerve stimulation

We employed the NEMOS tVNS device developed by Cerbomed (Erlangen, Germany), which was delivered with a pulse width of 200–300 μs at 25 Hz and an on–off cycle of 30 s. Two titan electrodes located in a structure similar to an earphone are placed on the cymba conchae of the left ear, an area thought to be exclusively innervated by the auricular branch of the vagus nerve [24].

#### Cardiac vagal activity

To assess cardiac vagal activity, we used the Faros 180° device from Mega Electronics (Kuopio, Finland) with a set sampling rate of 500 Hz. This device enables users to measure the electrocardiogram (ECG) signal as recommended by guidelines on HRV measurement [33]. We placed two disposable ECG pre-gelled electrodes (Ambu L-00-S/25, Ambu GmbH, Bad Nauheim, Germany) on the body, the positive electrode on the right infraclavicular fossa and the negative one on the left anterior axillary line below the 12^th^ rib.

The root mean square of successive differences (RMSSD) was chosen as indicator of cardiac vagal activity in the main analyses, with an increase of RMSSD meaning an increased cardiac vagal activity [12]. From ECG recordings we extracted HRV with Kubios software (University of Eastern Finland, Kuopio, Finland), visually inspected the full ECG recording, and manually removed artifacts [33]. Following recommendations for HRV measurement [12], we analyzed measurements in blocks of 5 min.

#### Subjective stimulation perception

At the end of each stimulation on–off cycle, that is, during 30-s breaks following 30-s stimulation units, the participants were instructed to answer questions on a Likert scale of 0 to 9 that were explicitly related to the past 30-s stimulation. The questions aimed at measuring perceived stimulation intensity and stimulation unpleasantness, respectively, and are “how intense is the tingle in your ear?” (0 = *I do not feel anything*, 9 = *extremely strong*) and “how pleasant or unpleasant is the sensation now?” (0 = *very pleasant*, 9 = *very unpleasant)*.

### Procedure

**Fig 1:**
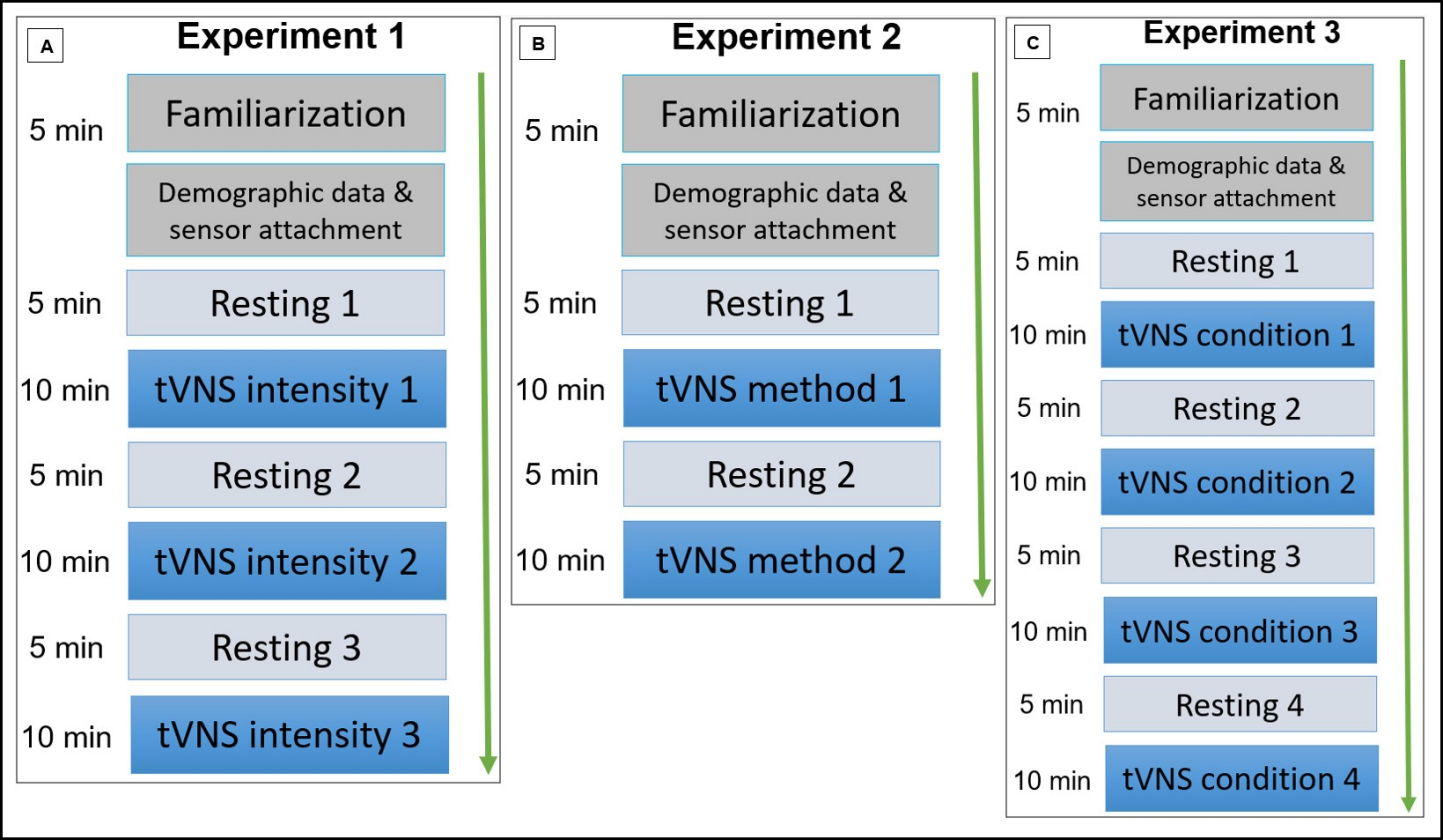
Study protocols for all experiments.

For each experiment, all participants underwent all stimulation conditions within one session in a counterbalanced order, to cancel out order effects. The participants were randomly assigned to the different possible order sequences. At the beginning of each experiment, a tVNS familiarization took place. In this phase, the participants received tVNS with 0.1 mA at the beginning and were instructed to increase the stimulation intensity within 5 min until reaching 1.0 mA. Following the familiarization period, HRV measurement was performed for all conditions. Each condition was measured within a block that consisted of two sub-blocks: The first one was performed to take resting cardiac vagal activity into account (5-min measuring interval), and the second one to measure cardiac vagal activity during the stimulation phase (10-min period, see Fig 1).

### Data analysis

Outliers (less than 1% of the data) were Winsorized, meaning that values higher/lower than two standard deviations from the mean were transformed into a value of two standard deviations from the mean. Since the HRV data were afterwards still not normally distributed, they were log-transformed to obtain a normal distribution, as is usually done in HRV research [33]. A repeated measures ANOVA was conducted on RMSSD as a dependent variable and included the independent variables time (resting, the first 5 min of the stimulation period (stimulation first half), and the last 5 min of the stimulation period (stimulation second half) and condition (stimulation intensities and stimulation methods or stimulation conditions, dependent on the experiment) as within-subject factors. Greenhouse–Geisser correction was used when sphericity was violated. In the case of a significant main or interaction effect, post hoc *t*-tests with aggregated means were conducted using Bonferroni correction. To quantify evidence for the hypotheses found, we ran Bayesian statistics using Bayesian information criteria [42] for the main analysis of RMSSD. Terms used to discuss the reported Bayes factors are based on Wetzels and colleagues’ recommendations [43] and have the following meanings: anecdotal or worth no more than a bare mention (0.333 < B_10_ < 3), substantial (0.100 < B_10_ ≤ 0.333 or 3 ≤ B_10_ < 10), strong (0.033 < B_10_ ≤ 0.100 or 10 < B_10_ < 30), very strong (0.010 < B_10_ ≤ 0.033 or 30 ≤ B_10_ < 100), and decisive (B_10_ ≤ 0.010 or B_10_ ≥ 100) evidence. Additionally, a Friedman test was performed for perceived intensity (mean) and for stimulation unpleasantness (mean) with condition (stimulation intensities or stimulation methods, dependent on the experiment) as a nonnormally distributed within-subject factor, with Wilcoxon tests for post hoc analyses. RMSSD was correlated using Spearman correlations with average perceived intensity and average stimulation unpleasantness, as well as average stimulation intensity during the free stimulation and the free sham conditions. To control for carry-over effects on RMSSD which potentially arose in the stimulation condition due to the previous stimulation condition, we tested the effect of position (i.e., first, second and third resting blocks in Experiment 1, first and second resting blocks in Experiment 2 and first, second, third and fourth resting blocks in Experiment 3) in one-way repeated measures ANOVAs or a paired *t*-test (for Experiment 2). Further, despite the fact that LF/HF and HR do not reflect clear physiological mechanisms regarding the functioning of the autonomic nervous system [30,31,44], for reasons of comparison with previous studies addressing effects of tVNS on HRV parameters, we also carried out three repeated measures MANOVA with RMSSD, LF/HF and HR as dependent variables. More details and the analyses can be found as supporting information (S1 Text). We used IBM SPSS Statistics 25 for data preparation and nonparametric analyses and JASP 0.9.1.0 to analyze the data for repeated measures ANOVAs, correlations, *t*-tests and Bayesian statistics. Significance level was α = .05.

## Experiment 1

In Experiment 1, we investigated set stimulation intensity by comparing three previously set stimulation intensities (0.5, 1.0, and 1.5 mA) with the aim of choosing one fixed stimulation intensity to be used in Experiments 2 and 3. These stimulation intensities were chosen following a previous study [38] on the effect of invasive cervical vagus nerve stimulation on recognition memory in humans using 0.5, 0.75, and 1.5 mA as stimulation intensities. To investigate the hypothesis related to the inverted U-shaped relationship with equal intervals, facilitating the results interpretation, we changed the intermediate stimulation intensity to 1.0 mA.

Besides expecting cardiac vagal activity to increase during tVNS compared to baseline measurements (H1), specifically for Experiment 1 we hypothesized (H2) that 1.0 mA would provoke the highest cardiac vagal activity when compared to 0.5 and 1.5 mA. In addition, we hypothesized (H2.1) that 1.5 mA would be perceived as the most uncomfortable stimulation intensity among the three intensities we deployed. Therefore, both (H2.2) the perception of stimulation intensity and (H2.3) the reported sensation of unpleasantness during 1.5-mA stimulation would be associated with a decrease in RMSSD during this condition. After excluding participants because of the excluding criteria and for technical issues during measurement, 61 participants were included in the data analysis (16 females, *M*_age_ = 23.32 years). We ran a repeated measures ANOVA for RMSSD with time (resting, stimulation first half, and stimulation second half) and stimulation intensity (0.5, 1.0 and 1.5 mA) as factors. For subjective ratings, a Friedman test was run for perceived intensity (median) and for stimulation unpleasantness (median) with condition (0.5, 1.0 and 1.5 mA) as a nonnormally distributed within-subject factor, with Wilcoxon tests for post hoc analyses.

### Results

#### Cardiac vagal activity

**Fig 2:**
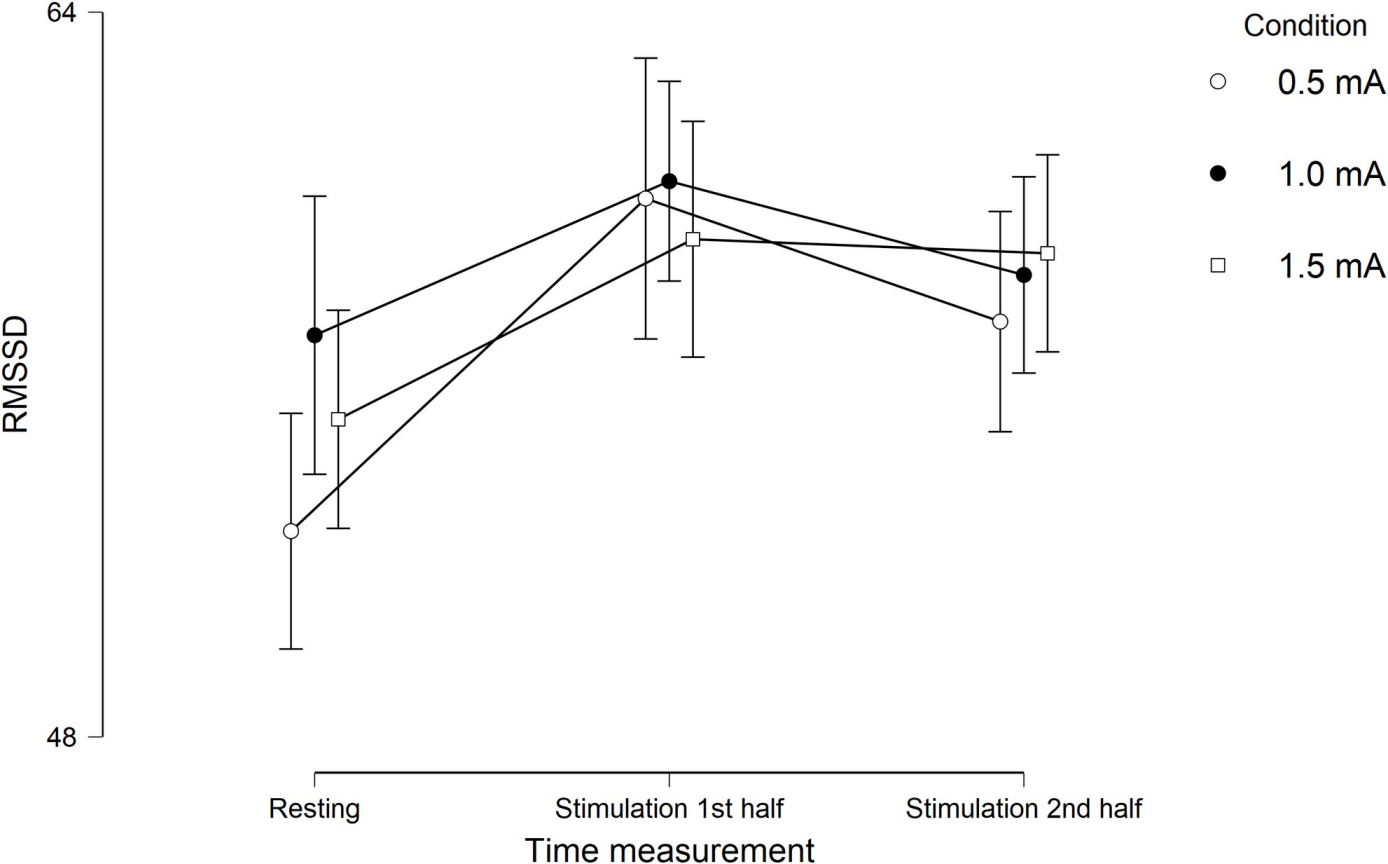
Experiment 1. **Mean scores of root mean square of successive differences (RMSSD).** Scores during different stimulation intensities at three time measurement points. Error bars represent confidence intervals (95%).

Descriptive statistics for RMSSD and for the subjective variables are presented in Table 1 and results of the hypothesis testing can be found in Table 2. A repeated measures ANOVA showed a main effect of measurement time for RMSSD, *F*(1.565, 93.900) = 8.590, *p* = .001, η_p_² = .125 (Fig 2). Three post hoc analyses were done, resulting in a *p* value of .017 after Bonferroni correction. They pointed out a significant increase from resting RMSSD (*M* = 54.81, *SD* = 26.86) to RMSSD during the first half of the stimulation period (*M* = 59.71, *SD* = 27.59), *t*(60) = 3.277, *p* = .002, *d* = 0.420, and no significant difference between RMSSD during the first and during the second half of the stimulation (*M* = 58.01, *SD* = 26.69), *t*(60) = 1.706, *p* = .093, or between resting RMSSD and RMSSD during the second half of the stimulation period, *t*(60) = 2.134, *p* = .037. No significant main effect of stimulation condition, *F*(2, 120) = 1.373, *p* = .257, or interaction effect of time × condition, *F*(3.507, 210.392) = 1.840, *p* = .131, for RMSSD was found. Because of the lack of a main effect of condition and the lack of an interaction between time and condition for RMSSD, we ran a Bayesian repeated-measures ANOVA. According to the estimated Bayes factors (alternative/null), data provided strong evidence against the alternative hypothesis for condition (B_10_ = 0.086) and for the interaction effect between condition and time (B_10_ = 0.045).

**Table 1:** Descriptive statistics for Experiments 1, 2 and 3. Mean scores and standard deviations for the root mean square of successive differences (RMSSD) and median values for perceived stimulation intensity and unpleasantness.

	Experiment 1			Experiment 2		Experiment 3			
	0.5 mA	1.0 mA	1.5 mA	Set stimulation	Free stimulation	Set active stimulation	Set sham stimulation	Free active stimulation	Free sham stimulation
**RMSSD values**									
Resting RMSSD	54.94 (26.39)	56.87 (29.67)	55.01 (25.98)	43.02 (16.74)	43.85 (17.46)	62.70 (24.37)	62.12 (26.97)	60.75 (24.33)	61.01 (25.65)
Stimulation 1^st^ half RMSSD	59.89 (29.20)	60.27 (30.17)	58.99 (26.88)	45.79 (18.55)	48.54 (22.41)	63.18 (26.67)	64.87 (28.71)	63.50 (25.65)	63.61 (27.99)
Stimulation 2^nd^ half RMSSD	57.17 (26.78)	58.20 (29.27)	58.68 (26.16)	46.73 (18.37)	48.44 (20.01)	65.79 (29.05)	66.21 (30.76)	64.72 (27.32)	64.04 (27.46)
**Subjective ratings**									
Perceived stimulation intensity	1.66 (1.05)	3.40 (2.06)	5.15 (2.19)	3.76 (2.47)	6.44 (2.29)	1.77 (2.32)	1.96 (2.54)	6.50 (2.00)	6.22 (1.83)
Unpleasantness	2.03 (1.91)	3.32 (2.06)	4.69 (2.24)	4.89 (2.76)	5.19 (2.55)	1.46 (1.93)	1.98 (2.02)	3.94 (2.21)	3.13 (2.05)

**Table 2:** Results of repeated measures analysis of variance for the root mean square of successive differences (RMSSD), with Bayesian analysis (B_10_) as well as post hoc tests for time measurement and for the subjective variables (Experiment 1).

**RMSSD**	***F*-value**	***p*-value**	**η**_**p**_**²**	**B**_**10**_
Time measurements	8.590	.001	.125	11,559.067
Stimulation condition	1.373	.257		0.086
Time x condition	1.840	.131		0.045
**Time measurements (RMSSD)**	***t*-value**	***p*-value**^**a**^	**Cohen’s *d***	**B**_**10**_
Resting vs. stimulation 1^st^ half	3.277	.002	.420	16.165
Stimulation 1^st^ half vs. 2^nd^ half	1.706	.093		0.548
Resting vs. stimulation 2^nd^ half	2.134	.037		1.153
**Perceived intensity**	***z*-value**	***p*-value**^**a**^	***r*-value**	**B**_**10**_
0.5 vs. 1.0 mA	6.630	< .001	.600	2.190*10^12^
0.5 vs. 1.5 mA	6.630	< .001	.388	1.048*10^17^
1.0 vs. 1.5 mA	4.290	< .001	.600	360,314
**Unpleasantness**				
0.5 vs. 1.0 mA	5.100	< .001	.461	1.245*10^6^
0.5 vs. 1.5 mA	5.590	< .001	.381	1.622*10^9^
**1.0 vs. 1.5 mA**	**4.210**	**< .001**	**.506**	**36,515**

^a^Bonferroni-corrected *p* = .017.

#### Estimation of carry-over effects on RMSSD

A univariate repeated measures ANOVA was performed to check if the position of the resting phases, namely the first (*M* = 54.92, *SD* = 29.43), the second (*M* = 54.83, *SD* = 26.83) and the third resting phase (*M* = 55.94, *SD* = 27.24), differed from each other regarding RMSSD. RMSSD during the different resting phases did not differ significantly from each other, *F*(2, 120) = 0.70, *p* = .498.

#### Subjective responses

A Friedman test indicated that the ratings of the stimulation intensities during the different stimulation conditions varied significantly across the different stimulation conditions, χ^2^(2) = 87.790, *p* < .001. Three post hoc analyses were done, resulting in a *p* value of .017 after Bonferroni correction. Post hoc Wilcoxon tests showed that the stimulation intensity 0.5 mA (*Mdn* = 1.73) was perceived as significantly lower than both 1.0 mA (*Mdn* = 3.24), *z* = 6.630, *p* < .001, *r* = .600, and 1.5 mA (*Mdn* = 4.856), *z* = 6.630, *p* < .001, *r* = .388. The stimulation intensity 1.5 mA was perceived as significantly stronger than 1.0 mA, *z* = 4.290, *p* < .001, *r* = .600.

In a similar vein, regarding unpleasantness, it was shown that the measures differed significantly across stimulation conditions, χ^2^(2) = 48.970, *p* < .001. Post hoc analyses with Wilcoxon tests (Bonferroni-corrected *p* = .017) indicated that the stimulation intensity 0.5 mA (*Mdn* = 2.05) was rated as significantly less unpleasant than both 1.0 mA (*Mdn* = 3.20), *z* = 5.100, *p* < .001, *r* = .461, and 1.5 mA (*Mdn* = 4.14), *z* = 5.590, *p* < .001, *r* = .381. The stimulation intensity 1.5 mA was perceived as significantly more unpleasant than 1.0 mA, *z* = 4.210, *p* < .001, *r* = .506. Moreover, perceived intensity correlated positively with RMSSD only during the 1.5 mA condition, during all three phases: resting (*r* = .331, *p* = .009), reactivity (*r* = .256, *p* = .047), and recovery (*r* = .307, *p* = .016). Unpleasantness correlated with no RMSSD measurements during stimulation.

### Discussion of Experiment 1

The aim of Experiment 1 was to compare the effect of three different stimulation intensities on physiological and subjective measurements. A significant increase of RMSSD during the stimulation phase was found compared to the resting phases prior to stimulation. H1 was therefore supported. However, this increase was general and thus not dependent on the different fixed stimulation intensities. These results were solidly supported by Bayesian statistics. H2, which was based on the Yerkes–Dodson principle [36], was therefore rejected. H2.1 was supported, since 1.5 mA was perceived as the strongest and most uncomfortable stimulation intensity among the three stimulation intensities. Finally, given that only perceived intensity correlated with RMSSD during the 1.5 mA condition and that this correlation was positive, H2.2 and H2.3 were rejected.

## Experiment 2

In Experiment 2, we compared the set stimulation method with the free stimulation method, in which the participants were instructed to freely choose a comfortable intensity just below the discomfort level. Given the absence of a statistical difference between the three stimulation intensities tested on cardiac vagal activity in Experiment 1, we chose the intermediate stimulation intensity from Experiment 1, 1.0 mA, as stimulation intensity for the set stimulation method in Experiment 2. Besides expecting cardiac vagal activity to increase during tVNS compared to baseline measurements (H1), for the reasons already stated, we hypothesized (H3) that the free stimulation method would evoke a higher increase in cardiac vagal activity compared to the set stimulation method. Moreover, we expected (H3.1) the set stimulation method, when compared to the free stimulation method, to be perceived as more uncomfortable method to set the stimulation intensity. Therefore, both (H3.2) the perception of stimulation intensity and (H3.3) the reported sensation of unpleasantness during set stimulation would be associated with a decrease in RMSSD during this condition.

The participants were aware of the chosen stimulation intensity over the course of the experiment in the free stimulation condition and could freely change the intensity once at the beginning of the on phases within the on–off cycles. After excluding participants because of the excluding criteria, 62 participants took part in Experiment 2 (26 females, *M*_age_ = 24.77 years) and the average chosen stimulation intensity in the free stimulation method was *M =* 1.78 mA (*SD* = 1.13). We ran a repeated measures ANOVA for RMSSD with time (resting, stimulation first half, and stimulation second half) and stimulation method (set stimulation method and free stimulation method) as factors. For subjective ratings, a *t*-test was run for perceived intensity (mean) and for stimulation unpleasantness (mean) with condition (set stimulation method and free stimulation method) as a normally distributed within-subject factor.

### Results

#### Cardiac vagal activity

**Fig 3:**
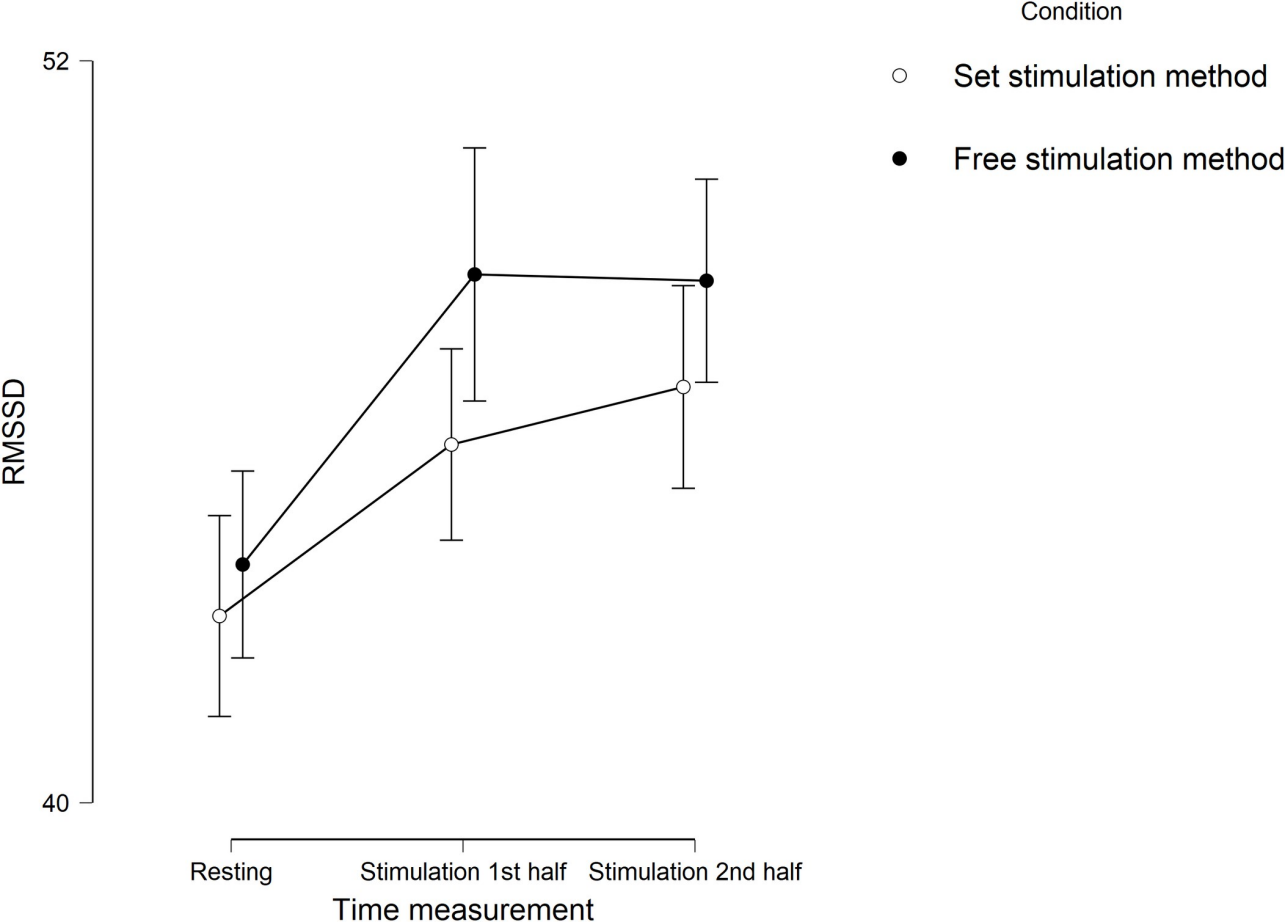
Experiment 2. **Mean scores of root mean square of successive differences (RMSSD)**. Scores during different stimulation methods at three time measurement points. Error bars represent confidence intervals (95%).

Descriptive statistics for RMSSD and for the subjective variables can be seen in Table 1 and results of the hypothesis testing can be found in Table 3. Regarding RMSSD, we found a main effect of time, *F*(2, 122) = 15.354, *p* < .001, η² = .206 (Fig 3). Three post hoc analyses were done, with pairwise comparisons (Bonferroni-corrected *p* = .017) showing a significant increase from resting RMSSD (*M* = 43.44, *SD* = 16.66) to RMSSD during the first half of the stimulation period (*M* = 44.82, *SD* = 17.39), *t*(59) = 2.960, *p* = .004, *d* = 0.382, and to RMSSD during the second half of the stimulation period (*M* = 45.29, *SD* = 17.47), *t*(59) = 3.410, *p* = .001, *d* = 0.440. RMSSD during stimulation first half was not significantly different from RMSSD during the second phase of the stimulation, *t*(59) = 0.935, *p* = .354. No significant main effect of condition, *F*(1, 61) = 1.715, *p* = .195, as well as no interaction effect of time × condition, *F*(1.811, 110.475) = 0.419, *p* = .888, for RMSSD was found. Bayes factors (alternative/null) provided substantial evidence against the main effect of condition (B_10_ = 0.129) and strong evidence against the interaction effect between condition and time (B_10_ = 0.060).

**Table 3:** Results of repeated measures analysis of variance for the root mean square of successive differences (RMSSD), with Bayesian analysis (B_10_), post hoc tests for time measurement and *t*-tests for the subjective variables (Experiment 2).

**RMSSD**	***F*-value**	***p*-value**	**η**_**p**_**²**	**B**_**10**_
Time measurements	15.354	< .001	0.206	1,777.357
Stimulation condition	1.715	.195		0.129
Time x condition	0.419	.888		0.060
**Time measurements (RMSSD)**	***t*-value**	***p*-value**^**a**^	**Cohen’s *d***	**B**_**10**_
Resting vs. stimulation 1^st^ half	2.960	.004	0.382	7.138
Stimulation 1^st^ half vs. 2^nd^ half	3.410	< .001	0.333	0.214
Resting vs. stimulation 2^nd^ half	0.935	.354		23.136
**Perceived intensity**	***t*-value**	***p*-value**^**a**^	**Cohen’s *d***	**B**_**10**_
Set stimulation vs. free stimulation	5.026	< .001	0.638	836.428
**Unpleasantness**				
**Set stimulation vs. free stimulation**	**0.766**	**< .001**	**0.447**	**0.173**

^a^Bonferroni-corrected *p* = .017.

#### Estimation of carry-over effects on RMSSD

A paired *t*-test was run to check if the position of the resting phases differed from each other regarding RMSSD. RMSSD during the first (*M* = 44.22, *SD* = 17.86) and the second resting phase (*M* = 44.67, *SD* = 17.45) did not differ significantly from each other, *t*(62) = 0.78, *p* = .441.

#### Subjective responses

Perceived stimulation intensity during the free stimulation method (*M* = 6.44, *SD* = 2.29) was significantly higher than perceived intensity during the set stimulation method (*M* = 3.76, *SD* = 2.47), *t*(61) = 5.026, *p* < .001, *d* = 0.638, and the rated unpleasantness (free: *M* = 5.19, *SD* = 2.55; set: *M* = 4.89, *SD* = 2.76) was significantly higher during the free stimulation method, *t*(61) = 0.766, *p* < .001, *d* = 0.447. Neither perceived intensity nor unpleasantness correlated with any RMSSD measurements during stimulation.

#### Chosen intensity during the free stimulation method

According to the one-sample *t*-test, the mean stimulation intensity chosen during the free stimulation phase was significantly higher than that set for the set stimulation phase, *t*(59) = 5.365, *p* < .001, *d* = 0.693. The mean chosen stimulation intensity correlated positively with RMSSD during the free stimulation phase (*r*_*s*_ = .357, *p* = .004). A higher chosen intensity was also associated with a higher rating of unpleasantness, *r*_*s*_ = .583, *p* < .001.

### Discussion of Experiment 2

In Experiment 2, RMSSD values showed a significant overall increase during the stimulation phase compared to the resting phase, thus giving support to H1. Importantly, similar to the results in Experiment 1, none of the different stimulation conditions significantly differed from each other regarding RMSSD values, meaning that H3 had to be rejected. Contrary to our expectation, the free stimulation method was perceived as stronger and more unpleasant than the set stimulation method. H3.1, thus, had to be rejected. Since neither perceived intensity nor unpleasantness correlated with any RMSSD measurements during stimulation, H3.2 and H3.3 were also rejected. It is noteworthy that higher chosen intensities in the free stimulation condition were associated with higher RMSSD values.

Given the lack of differences between the stimulation conditions in Experiments 1 and 2, we ran a third experiment to better understand the relationship between tVNS and time measurements. Specifically, we were interested in investigating if the overall increase in RMSSD during the stimulation conditions is in fact due to the stimulation itself or a result of other unknown factors. For this reason, in Experiment 3 we used the active stimulation conditions from Experiment 2 and included a set sham stimulation condition as well as a free sham stimulation condition.

## Experiment 3

We again expected cardiac vagal activity to increase during tVNS compared to baseline measurements (H1), and the free stimulation method to evoke a higher increase in cardiac vagal activity compared to the set stimulation method (H3). Moreover, considering the results of Experiment 2, for Experiment 3 we hypothesized (H4) that both active stimulations would provoke higher cardiac vagal activity (measured via RMSSD) when compared to the sham conditions. Consequently, we expected tVNS to affect cardiac vagal activity regardless of the active stimulation method that was tested. Furthermore, based on the results for the subjective ratings in Experiment 2, we expected (H4.1) the free stimulation method to be perceived as the most intense and most uncomfortable method among both methods we deployed. Therefore, both (H4.2) the perception of stimulation intensity and (H4.3) the reported sensation of unpleasantness during free stimulation would be associated with a decrease in RMSSD during this condition.

We ran a repeated measures ANOVA for RMSSD with time (resting, stimulation first half, and stimulation second half), stimulation method (set stimulation method and free stimulation method) and stimulation condition (active and sham stimulation) as factors. For subjective ratings, a Friedman test was run for perceived intensity (median) and for stimulation unpleasantness (median) with condition (set active stimulation, set sham stimulation, free active stimulation, free sham stimulation) as a nonnormally distributed within-subject factor, with Wilcoxon tests for post hoc analyses. The average chosen stimulation intensity in the free active stimulation condition was *M =* 2.5 mA (*SD* = 0.93), whereas for free sham stimulation it was *M =* 2.76 mA (*SD* = 1.01). Sixty participants took part in Experiment 3 (31 females, *M*_age_ of 23.62 years).

### Results

#### Cardiac vagal activity

**Fig 4:**
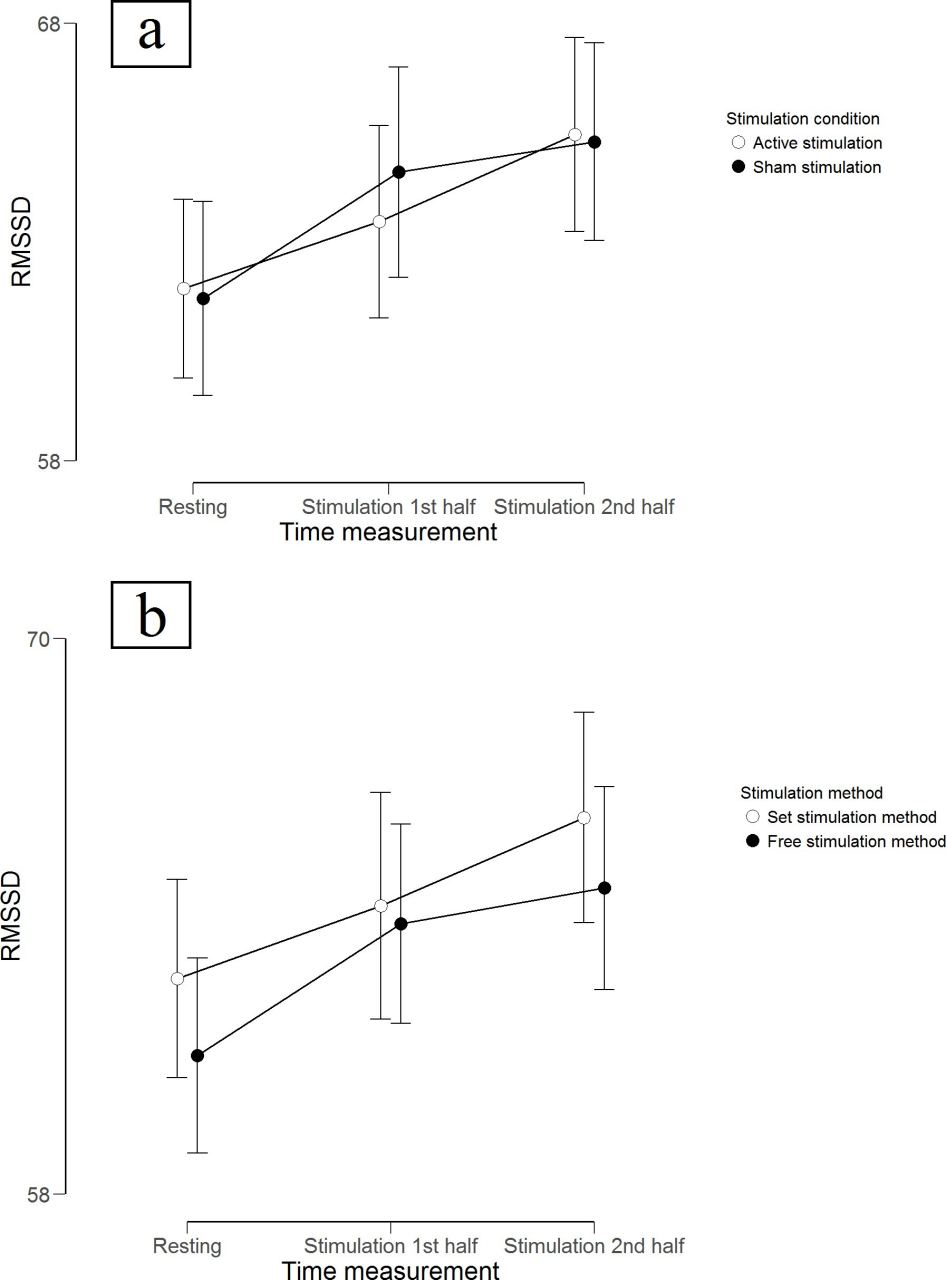
Experiment 3. **Mean scores of root mean square of successive differences (RMSSD).** Scores during (a) different stimulation conditions as well as (b) different stimulation methods at three time measurement points. Error bars represent confidence intervals (95%).

Descriptive statistics for RMSSD and for the subjective variables can be seen in Table 1 and results of the hypothesis testing can be found in Table 4. For RMSSD, a repeated measures ANOVA showed a main effect of time, *F*(2, 118) = 5.665, *p* = .004, η_p_² = .088. Pairwise comparisons (Bonferroni-corrected *p* = .017) showed a nonsignificant increase from resting RMSSD (*M* = 61.64, *SD* = 23.57) to RMSSD during the first half of the stimulation period (*M* = 63.79, *SD* = 25.20), *t*(59) = 2.172, *p* = .034, but a significant one to RMSSD in the second half (*M* = 65.19, *SD* = 26.90), *t*(59) = 3.080, *p* = .003, *d* = 0.398. RMSSD in the first half was not significantly different from RMSSD during the second half, *t*(59) = 1.350, *p* = .182. There was neither a main effect of stimulation condition, *F*(3, 177) = 0.031, *p* = .860 (Fig 4A), nor of stimulation method, *F*(3, 177) = 0.948, *p* = .334, and no interaction effect, *F*(4.766, 281.217) = 0.276, *p* = .759 (Fig 4B). Bayesian statistics were run for the null results and the estimated Bayes factors (alternative/null) strongly supported the lack of main effect for stimulation condition (B_10_ = 0.096), and substantially supported it for stimulation method (B_10_ = 0.149), and for the lack of interaction effect (B_10_ = 0.125).

**Table 4:** Results of repeated-measures analysis of variance for the root mean square of successive differences (RMSSD), with Bayesian analysis (B_10_) as well as post hoc tests for time measurement and for the subjective variables (Experiment 3).

**RMSSD**	***F*-value**	***p*-value**	**η_p_²**	**B**_**10**_
Stimulation condition	0.031	.860		0.096
Stimulation method	0.948	.334		0.149
Time measurements	5.665	.004	0.088	1.09
Condition x method x time	0.276	.759		0.125
**Time measurements (RMSSD)**	***t*-value**	***p*-value**^**a**^	**Cohen’s *d***	**B**_**10**_
Resting vs. stimulation 1^st^ half	2.172	.034		1.246
Resting vs. stimulation 2^nd^ half	3.080	.003	0.280	9.642
Stimulation 1^st^ half vs. 2^nd^ half	1.350	.182		.334
**Perceived intensity**	***z*-value**	***p*-value**^**b**^	***r*-value**	**B**_**10**_
Set active vs. set sham	0.184	.854		0.142
Set active vs. free active	5.740	< .001	0.741	1.140*10^8^
Set active vs free sham	6.570	< .001	0.848	4.458*10^12^
Set sham vs. free active	5.180	< .001	0.669	2.068*10^6^
Set sham vs. free sham	5.800	< .001	0.749	7.822*10^9^
Free active vs. free sham	0.501	.617		0.154
**Unpleasantness**				
Set active vs. set sham	0.532	.594		0.159
Set active vs. free active	4.040	< .001	0.520	386.451
Set active vs free sham	4.254	< .001	0.505	100.497
Set sham vs. free active	3.909	< .001	0.549	66.406
Set sham vs. free sham	3.823	< .001	0.494	274.774
**Free active vs. free sham**	**1.108**	**.268**		**0.184**

^a^Bonferroni-corrected *p* = .017.

^b^Bonferroni-corrected *p* = .008.

#### Estimation of carry-over effects on RMSSD

A univariate repeated measures ANOVA was run to check if the position of the resting phases differed from each other regarding RMSSD. Overall, RMSSD during the different resting phases differed significantly from each other, *F*(2.578, 152.125) = 16.020, *p* < .001, η_p_² = 0.214. Six post hoc analyses were done (Bonferroni-corrected *p* = .008). No significant difference between the resting RMSSD values in the first and the second positions could be found, *t*(59) = 0.999, *p* = .322. However, we found a significant increase from resting RMSSD in the first position, i.e. the resting phase before the first condition (*M* = 56.77, *SD* = 23.94), to resting RMSSD in the third position (*M* = 66.04, *SD* = 29.12), *t*(59) = 4.313, *p* < .001, *d* = 0.557, and to resting RMSSD in the fourth position (*M* = 67.83, *SD* = 29.94), *t*(59) = 5.005, *p* < .001, *d* = 0.646. Resting RMSSD increased significantly from the second position (*M* = 57.50, *SD* = 22.10) to the third one, *t*(59) = 3.814, *p* < .001, *d* = 0.492, and to the fourth one, *t*(59) = 5.125, *p* < .001, *d* = 0.662. No significant difference between the resting RMSSD values in the third and the fourth positions could be found, *t*(59) = 1.077, *p* = .286.

#### Subjective responses

With respect to subjective responses, a Friedman test indicated that the ratings of the stimulation intensities during the different stimulation conditions were significantly different, χ^2^(3) = 81.34, *p* < .001. Six post hoc analyses (*p* = .008 after Bonferroni correction) showed that set active stimulation (*Mdn* = 1.77) was rated as significantly weaker than free active stimulation (*Mdn* = 6.50), *z* = 5.74, *p* < .001, *r* = .741, and free sham stimulation (*Mdn* = 6.22), *z* = 6.57, *p* < .001, *r* = .848. Set sham stimulation (*Mdn* = 1.96) was perceived as significantly weaker than free active stimulation, *z* = 5.18, *p* < .001, *r* = .669, and free sham stimulation, *z* = 5.80, *p* < .001, *r* = .749. Perceived intensity correlated with no RMSSD measurements during stimulation.

In a similar vein, regarding unpleasantness, it was shown that participants in the stimulation conditions differed significantly in their estimations, χ^2^(3) = 29.030, *p* < .001. Post hoc analyses with Wilcoxon tests (Bonferroni-corrected *p* = .008) indicated that free active stimulation (*Mdn* = 3.94) was rated as significantly more unpleasant than set active stimulation (*Mdn* = 1.46), *z* = 4.03, *p* < .001, *r* = .520, and set sham stimulation (*Mdn* = 1.98), *z* = 3.91, *p* < .001, *r* = .505. Free sham stimulation (*Mdn* = 3.133) was perceived as significantly more unpleasant than set active stimulation, *z* = 4.25, *p* < .001, *r* = .549, and set sham stimulation, *z* = 3.82, *p* < .001, *r* = .493. Perceived intensity and unpleasantness correlated with no RMSSD measurements during stimulation.

#### Chosen intensity during the free stimulation method

Two one-sample *t*-tests (Bonferroni-corrected *p* = .025) revealed that the mean stimulation intensity chosen during the free active stimulation phase was significantly higher than that set for the set active stimulation phase, *t*(59) = 12.141, *p* < .001, *d* = 1.567, and that the mean stimulation intensity chosen during the free sham stimulation phase was significantly higher than that set for the set sham stimulation phase, *t*(59) = 13.542, *p* < .001, *d* = 1.748. The mean stimulation intensity chosen during the free active stimulation phase was significantly lower than that chosen during the free sham stimulation phase, *t*(59) = 2.501, *p* = .015, *d* = 0.323. Chosen stimulation intensity was not correlated with RMSSD during the free active and free sham stimulation phases. Likewise, chosen intensity in both free stimulation conditions was not correlated with perceived stimulation intensity and with the rating of unpleasantness in the respective phases.

### Discussion of Experiment 3

In line with Experiments 1 and 2, in Experiment 3 we found an increase from resting RMSSD to the RMSSD values during the stimulation phase, thus giving support to H1. Against H3, the effects of different stimulation methods on RMSSD did not differ from each other. Surprisingly, there was no difference between the active and sham conditions, meaning that H4 had to be rejected. Moreover, contrary to Experiments 1 and 2, there was evidence for a carry-over effect between the second and the third condition. The rated strength of the stimulation intensities within the stimulation conditions corresponded to the real stimulation intensities. In line with our expectations (H4.1), the stimulation intensities that could be chosen freely were perceived as higher than the set stimulation intensity 1.0 mA and were also rated as more unpleasant. Contrary to the results in Experiment 2 and to our expectations, in Experiment 3 the perception of stimulation intensity (H4.2) and the reported sensation of unpleasantness (H4.2) during the free stimulation condition were not associated with higher RMSSD values.

## General discussion

We investigated the influence of tVNS on cardiac vagal activity by testing the effect of different stimulation intensities on a vagally-mediated HRV parameter as well as on subjective ratings. This was the first study on tVNS to systematically investigate the effects of different stimulation intensities and different methods to determine them on cardiac vagal activity. The outcomes of the three experiments regarding effects of tVNS on cardiac vagal activity followed the same pattern, namely an increase in cardiac vagal activity from resting to at least one of the stimulation phases. However, this increase was general and thus not dependent on the different stimulation intensities or methods used to determine them, including the comparison between active and sham stimulation. This pattern of results was solidly supported by Bayesian estimations. On the subjective level, higher stimulation intensities were perceived as stronger, and the free stimulation method was perceived as stronger than the set stimulation method (Experiments 2 and 3). A stronger stimulation was generally also perceived as more unpleasant. Only in Experiment 1 a subjective rating of stimulation intensity or method correlated with RMSSD, namely the rating during the 1.5 mA-condition. Importantly, in Experiment 3 we found no differences between active and sham stimulations regarding cardiac vagal activity and this is in accordance with previous research [27–29,45]. Besides a rather unlikely potential placebo effect due to the single-blind design, we can think of two further possible explanations for this lack of differences. First, it is possible that stimulation parameters of tVNS do not affect its underlying mechanisms of action. In pain research, it has been speculated that VNS could send non-specific signals at the brainstem level. According to this idea, these signals would compete with incoming pain stimuli or alternatively trigger non-specific reflexes that activate pain inhibition, for instance by means of release of inhibitory neurotransmitters [46]. If this is also the case for the vagal pathway that is responsible for affecting cardiac activity, the effect of tVNS would be independent of its stimulation parameters (including the differentiation between active and sham stimulation), as long as the electrical signal evoked by tVNS reaches the brainstem nuclei involved.

Second, the suitability of the earlobe as a sham condition has been questioned. Peuker and Filler [7] offered a detailed description of the nerve distribution of different innervation areas of the human auricle, showing that the earlobe is free from vagal innervation and has the great auricular nerve as its only source of innervation. However, their findings were based on a sample of only seven human cadavers and in general, they also lacked substantial evidence that electrical stimulation on the earlobe cannot stimulate brain center nuclei that trigger an increase in cardiac vagal outflow [47]. Different fMRI studies [24,25,48] found evidence of activation of vagal pathways during tVNS when compared to earlobe stimulation; however, active stimulation was not always applied on the left cymba conchae. Furthermore, stimulation parameters such as the on–off cycles, differed partially from those in the present study [25]. This showcases once again that the lack of standards is an issue in tVNS research. Future studies should systematically address this topic by comparing areas that are currently being used as stimulation areas on the ear, such as the cymba conchae, outer auditory canal, and tragus, with stimulation on the earlobe using a multi-method approach—that is, taking behavioral and physiological measures into account.

Third, despite an increase of cardiac vagal activity from before to during tVNS and given that other reasons for this increase besides the action of tVNS cannot be completely ruled out, it is possible that RMSSD, as a marker of cardiac vagal activity, is not sensitive to afferent vagal changes triggered by tVNS. Since the tVNS signal on the left auricular branch of the vagus nerve is afferently sent to the prefrontal cortex, from where the signal is expected to go efferently to the heart [49], it is so far not clear if the electrical signal produced by tVNS is strong enough to overcome body-related barriers such as skin and blood vessels and therefore trigger vagal afferent firing in a way that would robustly increase prefrontal activity. This question may also be related to individual differences that can be relevant regarding electrical stimulation: Skin properties such as impedance, water content, structure, and subcutaneous fat thickness for instance have been shown to have an influence on the efficacy of transcutaneous electrical stimulation [50,51] and may have an influence on the response to tVNS. For this reason, to increase explained variance of the results, we recommend that future studies control for skin characteristics as well as further anatomical individual differences in the ear–fat thickness, for instance, is often measured using ultrasonography [50]. Some of these interindividual differences concerning skin characteristics can be related to age; specifically, older age is related to dryer, thinner and less hydrated skin and this can influence the sensibility to electrical transcutaneous stimulation [52]. Therefore, in case the sample consists of a heterogeneous population (in contrast to student samples), it is reasonable to take this potential confounder into account in the data analyses. Future studies should also address some of these questions by testing tVNS in combination with HRV and neuroimaging techniques such as fMRI and functional near-infrared spectroscopy (fNIRS), especially when it comes to investigating the sensitivity of cardiac vagal activity as a marker of vagal activation through tVNS. In these studies, a double-blind design should be considered to avoid possible placebo effects occurring with the sham stimulations.

Another noteworthy finding in the present study is that the stimulation intensities that were chosen on average in the free stimulation method were higher than the intensity normally reported in the tVNS literature [24,27,47]. A possible explanation is that most participants in the sample were sport students, which may have led to a sample with more participants with an inclination to be competitive. Competitiveness may explain why the participants often tended to choose stimulation intensities that were reported as uncomfortable without the existence of an apparent reward in the present study. Future studies should investigate the influence of different participants’ backgrounds on the outcomes related to tVNS and also use more standardized methods to determine the individual stimulation intensity, similar to other recent studies with tVNS [17,18].

Considering the previous literature on VNS [53], we expected the effects of short-term tVNS to be transient and to be confined to the stimulation periods. However, although analyses of carry-over effects on RMSSD at the resting phases provided evidence against the existence of carry-over effects in Experiments 1 and 2, an evidence for a carry-over effect in Experiment 3 was found. This interpretation comes from the results that the resting positions in Experiments 1 and 2 did not show any increase regarding RMSSD, whereas there was a significant increase between the first two resting phases and the last two resting phases in Experiment 3. Since Experiment 3 was the longest one among the three experiments and had the highest number of conditions (and consequently of stimulation periods), it is possible that a longer or more frequent stimulation plays a role when it comes to cardiac vagal effects of tVNS. Thus, the question related to the duration of tVNS effects still has to be further investigated. In a previous study, no relation was found between order of condition and HRV levels in the context of an experiment that lasted about 65 minutes, although it is not clear how exactly this was evaluated [27]. Nonetheless, it should be highlighted that the idea that tVNS has a transient effect comes from a previous VNS study [53] in which first, brain norepinephrine concentrations were measured and not cardiac vagal activity and second, the authors used (cervical) invasive vagus nerve stimulation in rats. Concerning tVNS, so far there is scarce evidence that a majority of brain areas that are thought to be part of the vagal pathway remain active after cessation of the stimulation of the left cymba conchae, although some areas continue to be active up to 11 minutes after stimulation [24]. Even though this finding lacks any inferential statistical analysis, it shows the importance of systematically taking into account the duration of effects after the stimulation period before drawing conclusions. Whether the effects of tVNS are transient is a question that should be specifically addressed by systematically comparing different time settings and stimulating the auricular branch of the vagus nerve, using cardiac vagal activity as a dependent variable and comparing its measurement before, during, and after the stimulation.

The same is valid for the often-discussed idea that the mechanism behind tVNS is related to the Yerkes–Dodson principle [36]: no evidence towards an inverted U-shaped curve could be found in the present study. Even if we used similar stimulation intensities as found in a previous study with humans receiving invasive VNS [38], it is important to highlight that this previous study investigated memory instead of cardiac vagal activity. Given the lack of outcome comparability, it is not possible to infer that the intensities that have been used correspond in fact to low, intermediary and high intensities, respectively. In the opposite direction, it is also possible that all intensities, especially in Experiment 1, are situated in the first, upward phase of the inverted U-shaped curve. Consequently, given this pattern could not be observed in the present study, its existence regarding the relationship between tVNS and cardiac vagal activity remains speculative. Future studies should further investigate this important issue by considering the points raised above and also address other domains that are thought to be modulated by tVNS, such as physiological (e.g. pupillary responses [54,55]) and cognitive processes (e.g. working memory and inhibitory control [3]).

### Limitations

The main limitation of our study was that we did not to take different physiological markers into account to compare them with cardiac vagal activity measurements, nor used neuroimaging techniques such as fMRI or fNIRS. Possible additional markers could be measurements of neurotransmitters thought to be linked to tVNS, for example, salivary alpha amylase, pupillary responses or P300 for norepinephrine release [18,56], or markers for sympathetic activity such as preejection period [57] and muscle sympathetic nerve activity [26]. Further, choosing to test all conditions in the same session may have led to some carry-over effects, as stated above. Moreover, respiration was not measured. Effects on RMSSD may be driven by influences of tVNS upon respiration, meaning that if an intervention can make persons breathe more slowly, RMSSD might in turn be increased [58]. Regarding the subjective responses, the formulation of the questions may have influenced the responses, since the question for measuring perceived intensity described a specific sensation provoked by tVNS (a tingle in the ear) whereas the unpleasantness question does not use such descriptive word for the stimulation sensation. Finally, although this was not the focus of the present study, a limitation may be that we did not take a cognitive paradigm into account, which would have enabled us to investigate the role of tVNS compared to sham stimulation as a moderator of cognitive functioning.

## Conclusions

The present study was the first attempt to systematically investigate, at the physiological and subjective level, the effects of different tVNS stimulation intensities as well as different stimulation methods. Based on the results summarized above, further investigation is needed on the potential effect of tVNS on cardiac vagal activity. Furthermore, the findings reported here revealed the importance of performing Bayesian analyses in addition to classical inferential hypothesis testing. Finally, this study pointed out the challenges in current research on tVNS and the need to address the lack of standards for stimulation to be used in cognitive and physiological studies.

## Supporting information

S1 Text Analysis of further HRV parameters and comparison with results of previous studies. (DOCX)
