# Supplementary material for: Influence of transcutaneous vagus nerve stimulation on cardiac vagal activity: Not different from sham stimulation and no effect of stimulation intensity
Source: PLoS One. 2019 Oct 11;14(10):e0223848. doi: 10.1371/journal.pone.0223848 (PMC6788680; doi:10.1371/journal.pone.0223848)
Supplement: S1 Text — (DOCX) [file pone.0223848.s001.docx]

**Supporting Information**

**S1 Text. Analysis of further HRV parameters and comparison with results of previous studies.**

We ran three repeated measures multivariate analyses of variance (rmMANOVAs), one for each experiment, with condition (stimulation intensities or stimulation methods, dependent on the experiment) as well as time measurement (resting phase, the first 5 min of the stimulation period (stimulation first half), and the last 5 min of the stimulation period (stimulation second half)) and with HR, RMSSD and LF/HF as dependent variables.

In Experiment 1, a rmMANOVA showed that there is a statistically significant difference in the HRV parameters regarding time measurements, Wilk's Λ = .750, *F*(6, 236) = 3.080, *p* < .001, η_p_² = .134, but not regarding different stimulation intensities, Wilk's Λ = .915, *F*(6, 236) = 1.792, *p* = .101. In follow-up univariate ANOVAs with Greenhouse–Geisser correction, there was a main effect in time measurement for HR, *F*(1.403, 84.184) = 3.694, *p* = .044, η_p_² = .058; for RMSSD, *F*(1.565, 84.184) = 8.590, *p* = .001, η_p_² = .125, and for LF/HF ratio, *F*(1.320, 84.184) = 5.453, *p* = .014, η_p_² = .083. Six pairwise comparisons for each HRV parameter (Bonferroni-corrected *p* = .008) showed that HR significantly decreased between stimulation’s first half and second half (*p* < .001); RMSSD increased significantly from resting phase to the first half of the stimulation phase (*p* = .001) and also from the first to the second half (*p* = .008); LF/HF ratio increased significantly from resting to the second half of the stimulation phase (*p* = .006).

In Experiment 2, we also found only a main effect of time, Wilk's Λ = .634, *F*(6, 232) = 9.895, *p* < .001, η_p_² = .204. In follow-up univariate ANOVAs with Greenhouse–Geisser correction if necessary, there was a main effect in time measurement for HR, *F*(1.537, 90.662) = 21.552, *p* < .001, η_p_² = .268, for RMSSD, *F*(2, 118) = 15.354, *p* < .001, η_p_² = .206, but not for LF/HF ratio, *F*(1.684, 90.662) = 2.391, *p* = .106. Six pairwise comparisons for each HRV parameter (Bonferroni-corrected *p* = .008) showed that HR significantly decreased across all measurement time points (*p* < .001); RMSSD increased significantly from resting to first half (*p* = .001) and from resting to second half (*p* < .001).

Likewise, in Experiment 3 we also found a main effect of time, Wilk's Λ = .833, *F*(6, 232) = 3.710, *p* = .002, η_p_² = .088, with this main effect being found for HR, *F*(1.875, 110.616) = 10.401, *p* < .001, η_p_² = .150 and for RMSSD, *F*(1.868, 110.216) = 4.563, *p* = .014, η_p_² = .072, but not for LF/HF ratio, *F*(1.826, 107.748) = 1.221, *p* = .296. Pairwise comparisons revealed that, after Bonferroni-correcting *p* value (*p* = .008), there is a significant difference only for HR, namely a decrease from resting to second half of the stimulation (*p* < .001).

Taken together, tVNS exerted similar effects on the three HRV parameters, with only time measurement showing significant differences without showing any effect of stimulation intensities. However, only in Experiment 1 we found a significant main effect of time for LF/HF ratio, independently of the stimulation condition. On the one hand, in accordance with previous studies (6,26), we found a significant decrease in HR during active and sham tVNS. On the other hand, contrary to our results regarding LF/HF ratio, Clancy et al. (26) found a significant decrease in LF/HF ratio during active tVNS but no significant change in the sham group. Hence, we could observe the same results reported by Badran and colleagues (6) and partially replicate the results reported by Clancy and colleagues (26). Nonetheless, it is important to highlight that, as already stated in the manuscript, these authors used different stimulation parameters we did in the present study, namely different pulse-width, frequency, on–off cycle and, perhaps most importantly, they stimulated a different area of the ear, the tragus, which is thought to be innervated by different nerves as the cymba conchae (7), which we stimulated in our research project. For this reason, any comparison between results from these studies should be treated with considerable caution.
